# ‘Brain fog’, guilt, and gratitude: experiences of symptoms and life changes in older survivors 6 months after hospitalisation for COVID-19

**DOI:** 10.1007/s41999-022-00630-8

**Published:** 2022-03-10

**Authors:** Kristi E. Heiberg, Anne K. T. Heggestad, Nina Jøranson, Hilde Lausund, Grete Breievne, Marius Myrstad, Anette H. Ranhoff, Marte M. Walle-Hansen, Vigdis Bruun-Olsen

**Affiliations:** 1 Faculty of Health Science, Oslo Metropolitan University, Oslo, Norway; 2 Department of Medical Research, Bærum Hospital Vestre Viken Hospital Trust, Drammen, Norway; 3 Faculty of Health Studies, VID Specialized University, Oslo, Norway; 4 Faculty of Health and Social Sciences, University of South-Eastern Norway, Drammen, Norway; 5 Diakonhjemmet Hospital Trust, Oslo, Norway; 6 University of Bergen, Bergen, Norway

**Keywords:** COVID-19, Experience, Older adult, Symptoms, Salutogenesis, Qualitative

## Abstract

**Purpose:**

Several of those who have been infected with COVID-19 suffer from the post-COVID-19 condition months after the acute infection. Little is known about how older survivors have experienced the consequences and how these have affected their lives. The aim of this study was to explore how older survivors experienced post-COVID-19 condition and life changes approximately 6 months after hospitalisation for COVID-19.

**Methods:**

The study had an explorative and descriptive design. Semi-structured interviews were performed with 17 participants from two local hospitals, 11 men and 6 women, aged 60 years and older (age range 60–96), approximately 6 months after hospitalisation for COVID-19. A thematic descriptive analysis inspired by Braun and Clarke was used.

**Results:**

Two main themes and seven subthemes were revealed. The main themes were: *From few to various persistent symptoms* and *Existential thoughts and reflections.* Most of the participants experienced various physical and/or cognitive symptoms, such as reduced physical fitness, heavy breathing, fatigue, and ‘brain fog’. On the other hand, they also experienced guilt and gratitude for having survived. The recognition of having achieved other life perspectives was also present.

**Conclusion:**

Six months after undergone COVID-19 the participants still experienced various distressing symptoms, which were in line with larger studies. The novel findings of this study were connected to the existential area, where the patients’ thoughts and reflections of guilt, gratitude, and new life perspectives were revealed. These findings are important for health professionals to consider when treating patients after COVID-19.

## Key summary points

**Aim:**

The aim of the study is to explore older participants’ experiences of symptoms and life changes approximately 6 months after hospitalisation for COVID-19.

**Findings:**

The participants experienced various persistent physical and cognitive symptoms. At the same time, they experienced gratitude for having survived and for having achieved new life perspectives.

**Message:**

Despite suffering from the post-COVID-19 condition the older adults’ coping resources helped them achieve new life perspectives.

## Introduction

The first cases of Severe Acute Respiratory Syndrome Coronavirus 2 (SARS-CoV-2) were confirmed in China during the last weeks of 2019 (COVID-19) [1]. Although COVID-19 predominantly affects the respiratory system, it is a multisystem condition with varying clinical courses, from mild symptoms to death [2]. Older patients present additional symptoms of acute functional decline, confusion, and asthenia [3, 4]. As of February 2022, the World Health Organization (WHO) has reported more than 387 million confirmed cases and 5.7 million deaths, worldwide [5].

Today, approximately 2 years after the outbreak of the pandemic, more than 90% of the Norwegian population is vaccinated, but we are still grappling with new virus mutations, new infected persons, and additionally those that suffer from the post-COVID-19 condition [6, 7]. Post-COVID-19 condition is the term of the multisystem consequences of the virus that lasts for more than 3 months [7–9]. It appears to be a multisystem condition associated with a complex array of respiratory, cardiovascular, neurological, musculoskeletal, rheumatological, gastrointestinal, dermatological, and immunological symptoms. These symptoms vary in severity, frequency, and duration [10–12], and the pathophysiological mechanisms are not fully understood.

In a study of COVID-19 survivors aged 60 years and older who had been admitted to four general Norwegian hospitals, more than 50% reported a decrease in health-related quality of life (HR-QoL) 6 months after hospitalisation, measured by the EuroQol 5-dimensional-5 levels (EQ.5D5L) questionnaire. Approximately 30% reported impaired mobility and ability to carry out usual activities, and they had more pain and discomfort compared to before the acute infection [13]. Considering the similar clinical presentation of COVID-19 and other viral respiratory infections such as SARS, it may be possible that patients with COVID-19 will display similar impairments with incomplete recovery of physical function as long as 1–2 years post-infection [14]. Moreover, it may be assumed that the severity of the disease, with a strong inflammatory response, hypoxemia requiring long-term oxygen treatment, and prolonged bedrest could imply even more severe long-term consequences, especially for the old and frail.

Going through serious illness may change an individual’s life perspectives. Existential thoughts, i.e. thoughts concerning the meaning, purpose, and value of human existence [15] often affect people in such a life situation [16]. An individual’s resources to cope with negative experiences can be linked to Antonovsky’s salutogenic framework and the concept of sense of coherence. Sense of coherence is described as one’s ability to holistically comprehend a stressful experience, the capacity to use the available resources, and the enduring and dynamic feeling of confidence that the experiences in life are comprehensible, manageable, and meaningful [17]. The concept called ‘salutogenesis’ focuses on our innate capacity to create and maintain health and well-being in the face of adversity [18], and more on people’s resources than on pathogenesis [19]. Our internal healing resources and potential for active adaptation to new circumstances is emphasised in the salutogenic model [19]. An individual’s sense of coherence may play a role in sustaining health when affected with stressors, such as serious illness.

There have been published several qualitative studies on different topics related to COVID-19, for example on public perceptions and experiences of social distancing and social isolation [20]), and on the public’s psychological health during the pandemic outbreak [21]. Health professionals’ experiences of working with patients suffering from COVID-19 have also been explored [22, 23]. Furthermore, COVID-19 related stress and concerns of people with cancer [24] and with respiratory conditions [25] have been topics. Some have reported patients’ experiences of psychological disturbances during hospitalisation [26], when in isolation [27], and throughout the disease crisis [28]. Only a few prior qualitative studies have explored the patients’ experiences of suffering from the post-COVID-19 condition. One study documented the patients’ lived experiences of accessing and receiving healthcare in the prolonged phase [29]. Another study gave insight into the patients’ challenges of managing physical activity alongside the extended symptoms of the post-COVID-19 condition [30]. However, we have not identified qualitative studies exploring older patients’ experiences of living with post-COVID-19 condition and how their life perspectives may have changed 6 months after hospitalisation for severe infection. Older patients’ experiences with COVID-19 should be explored in greater depth to contribute to guide health professionals in their clinical meeting with these patients.

The aim of this study was, therefore, to explore how older participants aged 60 years and older experience post-COVID-19 condition and life changes 6 months after hospitalisation for severe COVID-19.

## Methods

### Design

An explorative and descriptive design was used. Semi-structured interviews were conducted in October/November 2020 to explore the participants’ experiences of COVID-19 and how life had changed approximately 6 months after hospitalisation during the first phase of the pandemic. This sub-study was part of a multi-centre cohort study in South-Eastern Norway and has been previously described in detail [13].

### Participants

Participants in the multi-centre study, aged 60 years and older came to a follow-up consultation 6 months after hospitalisation. Participants from two of the four hospitals in the multi-centre study were invited to participate in the current sub-study if they were considered physically and cognitively able to perform an interview. Only two of those invited refused to participate; one because of high age, and the other was staying far from the hospital. A written informed consent was obtained by all participants.

Seventeen participants (11 men and 6 women, age range 60–96 years) were interviewed in 14 in-depth interviews, as 3 of the interviews were performed with spouses in which both had been hospitalised with COVID-19 during the same period (Table 1).

**Table 1:** Characteristics of the participants

Participants (male/female)	Age (range)	Cohabitant	Days spent on mechanical ventilation	Length of hospital stay (days)
P1 (m)	70–74	Yes	31	42
P2 (m)	75–79	Yes		22
P3 (m)	95–99	No		14
P4 (m)	60–64	Yes	14	21
P5 (f)	75–79	No		3
P6 (f)	60–64	Yes		3
P7 (m)	70–74	No		21
P8 (m)	60–64	Yes		34
P9 (f)	65–69	Yes		5
P10 (f)	70–74	Yes		10
P11 (m)	70–74	Yes	10	28
P12 (m)	65–69	Yes		2
P13 (f)	85–89	Yes		3
P14 (m)	90–94	Yes		4
P15 (m)	80–84	Yes	12	19
P16 (f)	80–84	Yes	55	62
P17 (m)	65–69	Yes		9

### Data collection

We prepared a semi-structured interview guide beforehand. It contained questions about the participants’ perceptions of their recovery and how life had changed approximately 6 months after they were hospitalised due to COVID-19. Six of the authors (NJ/AKTH, GB/HL, KEH/VB-O), all female researchers working in pairs, performed the interviews. Their professions were nurses and physiotherapists, and they all had broad experience from both clinical work and research.

The prepared semi-structured interview guide contained open-ended questions aiming to get as rich descriptions as possible of the participants’ experiences. The interviews were performed either at the hospital, at the researchers’ office, or in the participants’ home. They lasted for 45–90 min and were audio-recorded. An external transcriptor transcribed the interviews into text.

### Data analysis

In analysing the data, we used a thematic descriptive analysis inspired by Braun and Clarke [31–33]. It is an independent qualitative descriptive approach used for identifying, analysing, and reporting patterns (themes) within textual data [34]. Additionally, the thematic analysis helps to organize and describe the data set in a fairly rich detail, and during the analysis various aspects of the research topic can be interpreted [31].

A realist method of the thematic analysis was applied. In the analytic process six steps were followed [31]. These steps were: (1) Familiarising with the data, (2) Generating initial codes, (3) Searching for themes, (4) Reviewing themes, (5) Defining and naming themes, and (6) Producing the report [31]. To accomplish deep reflection and engagement with the data the researchers were going back and forth between the steps described above [32].

First, we read the text separately several times (NJ, AKTH, GB, HL, VB-O, KEH) to obtain a global sense of the participants’ experiences. Thereafter, we agreed on the main essence of data. Our guiding question was: What do these data tell us about the participants’ experiences of suffering from late symptoms and life after COVID-19? The two main authors (KEH, VB-O) performed the last part of the analysis. After further reading through the data, we identified places in the text that were related to the participants’ experiences and reflections on suffering from the post-COVID-19 condition, the psychological aspects, and life perspectives. We identified excerpts from the text and coded them manually. The meaning of each excerpt was condensed into fewer words. Sub-themes were developed as we combined and grouped the codes. In the entire analytic process, there was a constant interplay between the various analytical steps. During the analytic process we (KEH, VB-O) reached consensus through discussions.

In qualitative research, a key aspect is to make the authors' preconceptions visible [35]. The two main authors (KEH, VB-O) were physiotherapists, and we believed that most of the survivors from COVID-19 would have exercised and regained their physical and cognitive function after 6 months. These preconceptions enabled us to challenge some of the interviewees' expressions and discuss them throughout the analytic process.

### Ethics

This study was a sub-study of a larger multi-centre observational cohort study which was planned in the beginning of the pandemic [13]. Ethical approval was granted by the Regional Research Committee in Eastern Norway (reference number 155425). Only participants who were able to give written informed consent and able to be interviewed were included in the study. They were assured confidentiality and that they could withdraw at any time without any consequences.

### Results

The thematic analysis yielded two main themes and seven subthemes. The main themes were: *From few to various persistent symptoms* and *Existential thoughts and reflections.* The corresponding subthemes are presented in the following section. We extracted excerpts from the transcribed text of the interviews to substantiate the subthemes. The excerpts are presented in italics. Quotation marks refer to the participants are presented in Table 1.

## Main theme 1: from few to various persistent symptoms

### Few persistent symptoms

A few participants experienced that they had completely recovered from the disease. Some had recovered fast, while others had needed a longer time to become free of symptoms. However, most of the participants experienced symptoms in line with post-COVID-19 condition. The symptoms varied in intensity and number. An older man started to say that he had none ongoing symptoms, but after some reflection, he realized that he still had physical problems:

*For me, late symptoms are relatively few, if there are any at all …. This thing with my lungs - bad breathing and so on - I can feel that, for sure. But whether it’s simply a question of more exercise or not, I don't know. (Male participant, P15)*

## Reduced physical fitness and heavy breathing

Many mentioned reduced physical fitness and lung symptoms, such as heavy breathing as persistent symptoms. They often explained their heavy breathing as related to reduced physical fitness. One of the eldest women said that even though she exercised regularly, she felt she was still not fit. When comparing to her previous walking ability, she now realized that she could only walk for shorter distances. For many it was a great challenge that they still experienced complex physical and cognitive symptoms. A woman described it in this way:

*The worst trauma has been that I’ve got a post-traumatic stress syndrome. I don't feel as strong as I did before. And I’ve got some trouble with my heart, and I feel different…. and I breathe more heavily*. *(Female participant, P9)*

## Fatigue

Fatigue was a common disabling symptom among the participants. To them fatigue was an unknown condition. It felt different from normal tiredness and was experienced as exhaustion:

*I'm getting more tired. I'm getting tired faster. (Male participant, P6)*

*What affects me now is the current situation with persistent symptoms that makes me tired quickly. I've learned a new word: Fatigue. When the battery is flat, it really is flat. (Male participant, P8)*

When ‘the battery is flat’, they experienced that they could handle less. Especially for those who were ambitious about being active, this felt awful and stressing. They also experienced to be more easily upset and vulnerable in relation towards family members and friends.

## Depression, anxiety, and cognitive symptoms

A few also expressed that their mental condition had been a challenge during the recovery period. They had suffered from depression and anxiety. One woman described the depression as ‘black octopus arms’ attempting to pull her down.

The participants’ ongoing symptoms were often related to different cognitive challenges. Several mentioned losses of memory, problems with understanding concepts, and finding the right words. Attention deficits, lack of ability to focus over time, and challenges in performing executive functions were also expressed. One participant who had been on a mechanical ventilator said that he was feeling indifferent and lethargic for a long time. He named it as a ‘brain fog’ that was slowly easing:

*I felt for quite a while a form of fatigue and indifference. …. I had a general lethargy that lasted, even though there were other improvements. There was such a feeling of ‘brain fog’ that was easing, slowly. (Male participant, P11)*

Some expressed that they were more easily stressed and unfocused. This was especially a challenge in terms of getting back to work. One participant, who was still on sick leave from work due to fatigue and cognitive symptoms, told that his head worked fine with respect to sit on the terrace and drink coffee. But when he tried to work at his own computer, read a document, process it, and come up with a solution or an answer, he was completely out of focus after only 15–20 min.

For others, it was more a feeling of not being completely ‘in place’ and no longer being quite the same person as before, as expressed by this participant:

*I'm still not quite fully present and I may not ever again become quite the person I once was. But then you just must accept it. (Female participant, P9)*

### Other symptoms

Many participants also experienced to suffer from various other symptoms of post-COVID-19 condition. These symptoms were musculoskeletal pain in the neck, shoulders and legs, headache, hair loss, and gained weight. Several also mentioned that they had been diagnosed with new diagnoses by their physician during the last 6 months, such as post-traumatic stress syndrome, polyneuropathy, increased blood pressure, atrial fibrillation, aortic valve insufficiency, osteoarthritis, gout, and skin cancer. Furthermore, some of the participants had suffered from serious complications in the acute phase, such as pneumonia, blood clots in the lungs, and heart attack.

## Main theme 2: existential thoughts and reflections

### Stigmatisation, guilt, and gratitude

Some of the participants had experienced that they were stigmatised due to their condition. They recognized that people around them were afraid and sceptical to be with them and that good friends avoided them. For some it was unclear for how long they were contagious and could infect others. However, 6 months after receiving the disease, a feeling of relief was present among many of the participants. When these interviews took place, no one was yet vaccinated. Consequently, only those who had undergone the disease were immune in a social context, and they felt relieved.

The participants had been infected with COVID-19 by someone else. Some could point out where and how this had happened, such as on the bus, on a domestic flight, while abroad, at home with family members, at a party, at work, or at the gym. One woman working in a nursing home expressed a great relief for not having transmitted the disease to anyone at the nursing home, even though she was at work the day before she got ill.

However, some participants had transmitted the disease to family and friends before they were aware that they were contagious. For this they felt guilt. One woman described how the feeling of guilt was mixed with the feeling of gratitude when her sister survived:

*I did feel guilty*. *But when she survived and recovered quickly, the relief was so great, and I was so grateful that I decided to become a new and better person! (Female participant, P5)*

For the survivors the gratitude for still being alive was great and a dominating feeling:

*We have reasons to be happy for still having each other. (Male participant, P12)*

*I’m so glad I’m alive! (Female participant, P9)*

*We’ve been lucky who survived. I’m very grateful (Male participant, P14)*

### A span between negative and positive life changes

Some of the participants were still working when they were infected with COVID-19. The thought of whether they would be able to continue their working career was a serious stressor. One participant had to reconsider his plans, in particular his plans of working for many more years:

*Yes, at night I fall asleep with the thoughts of my work situation, and I wake up in the morning with these thoughts... I try not to think about it - but I think about it all the time. Is this how the future will be? Is this how the new decade will become? (Male participant, P8)*

After undergoing serious illness and having survived, a change in life perspectives may appear. To some even to the better, as expressed in this way:

*Going through corona has changed my life for the better. When you're so far down, when you're seriously ill…..Then, when you recover from it you feel that life is not so bad after all. (Male participant, P2)*

Consequently, the idea of making some changes in life may occur:

*I want to become a more generous person. To invite more often, to be more outdoors, and so on. A little more like dancing in the sun, you know. (Female participant, P5)*

To many, a different life perspective was a positive effect from having survived. They felt that being alive should not be taken for granted and that the new experience could be used in future life:

*I can use this. Add it, as one can do with all experiences in life. Use them. Instead of focusing on the trauma, the amazing experience of being alive is very strong! (Female participant, P9)*

Existential thoughts may be a consequence of going through serious illness. During the acute phase, several of the participants said that they were afraid of dying. This was the worst trauma they had ever experienced. A woman described how she had started to reflect on life in a new way during her recovery process:

*This has made me wonder even more about this life. (Female participant, P9)*

Another woman expressed how strange and unbelievable it was that a pandemic should flood the western world, reminding us that we are a small piece of a great puzzle:

*You experience being a small piece in something so big, something global. And at the same time so scary. It becomes existentially challenging. The fact that we are all part of the same world. I became a part of it* [the pandemic], *me too. (Female participant, P10)*

## Discussion

This study indicated that many of the participants still experienced to suffer from the post-COVID-19 condition with several physical and cognitive symptoms, such as decreased physical fitness, heavy breathing, fatigue, and cognitive symptoms like ‘brain fog’ up till 6 months after hospitalisation. Despite the ongoing symptoms, they expressed guilt and gratitude for having survived, and a recognition of having achieved new life perspectives.

The participants in this study had all been seriously ill and hospitalised because of COVID-19. Approximately 1/3 of those interviewed had been treated on a mechanical ventilator for at mean 24 days. After receiving mechanical ventilation in ICU, prior research has shown that up to 80% of survivors experience long-lasting sequelae, such as new or worsened physical, cognitive, and/or mental health conditions, called the post-intensive care syndrome [36]. In our study, also several of those who had not needed treatment on mechanical ventilator reported to suffer from the post-COVID-19 condition.

Through the interviews many of our participants expressed that they suffered from both physical and cognitive symptoms approximately 6 months after discharge from hospital. Prior research has revealed that COVID-19 often brings a negative long-term impact on the patients’ lives with impaired HR-QoL, measured by questionnaires as endpoints [13, 21]. Knowledge drawn from the present interviews revealed examples of how impaired HR-QoL can be experienced. Several expressed that they suffered from a condition with cognitive dysfunction and memory loss, which was called ‘brain fog’. This feature is previously shown in other studies [30, 37]. The authors of an international online survey with 3762 respondents from 56 countries reported that 55% experienced ‘brain fog’ 7 months after onset of Covid-19 [10], indicating that ‘brain-fog’ is a common and long-lasting symptom. In addition, prior studies have revealed that approximately 1/3 of patients hospitalised due to COVID-19 during the first wave of the pandemic in Norway experienced delirium, showing highest prevalence among older patients [38, 39]. Delirium is associated with long-lasting cognitive symptoms in other patient groups [40]. The persistent cognitive symptoms described by our participants may be features of delirium sequela.

The present findings of various persistent symptoms were in line with findings in a recent review in which symptoms, complications, and management of the post-COVID-19 condition were reported [6]. Fatigue was a common symptom, and older age, female gender, hospital admission, and initial dyspnoea were among the risk factors that were significantly associated with an increased risk of developing persistent symptoms [6]. Additionally, several of our participants reported new and serious diagnoses, such as osteoarthritis, gout, increased blood pressure, and atrial fibrillation. These were not necessarily related to COVID-19, but still experienced as a heavy burden.

During the recovery period many participants had experienced a feeling of guilt for having had the disease and infecting their loved ones. Stressors may produce important implications on psychological outcomes in survivors. This was in accordance with a study describing that COVID-19 survivors often felt psychological traumas, such as guilt related to being a carrier [28]. Furthermore, an interview study with patients during hospital stay found that the patients felt guilt for their family members and others that were put in isolation and medical observation after they had been in close contact [26]. Survivors from the previous SARS epidemic also described stressors, such as being blamed by members from community and family for spreading illness, high death rates, the fear of infecting loved ones, and survivors’ guilt [41]. Guilt seemed, therefore, to be a common stressor after serious infectious disease.

Going through COVID-19 and the post-COVID-19 condition were also experienced by the participants as serious stressors. These stressors could be understood in line with Antonovsky’s description of human stress, involving somatic-, psychological-, and social aspects [17]. It is previously reported that severe COVID-19 affected the participants’ HR-QoL, with impaired mobility and ability to carry out activities of daily living 6 months after hospitalisation [13]. Stressors may have affected the participants’ HR-QoL in the direction of the unhealthy end of the health continuum [17], while coping and sense of coherence play a role in managing stressors and sustaining health [42].

In the present study several of the participants expressed gratitude for still being alive despite their ongoing symptoms. They told about their existential thoughts. Existential crises or traumatic experiences may activate existential thoughts and fears as people start to be aware of their own mortality [16]. Thereby a change in life perspectives may occur. The contradictory findings of still having various symptoms, but also the ability to cope with life, represented a huge step forward towards the healthy end of Antonovsky’s health continuum [17]. The findings illuminate the patients’ ability to engage resources to cope with stressors, such as the negative experiences. Framing our findings with the salutogenic model make us better understand the contradictory expressions of the participants suffering from the post-COVID-19 condition. This knowledge can be useful for health professionals and enable them to approach the older patients more holistically and better support their coping resources.

There are some strengths and weaknesses in this study. We preserved variability and reflexivity, and we established credibility by that all interviews were read by six of the authors (KEH, VB-O, AKTH, NJ, GB, HL). The data were analysed independently and together by the two main authors (KEH, VB-O). In the analytic process we followed the steps described by Braun and Clark [31]. To strengthen the validity of the findings we recruited a sample of participants from both genders and with wide age range. They had been hospitalised with severe COVID-19 in two different hospitals. The main authors’ preconceptions, gender, and profession are clarified to assure the credibility. This were according to the criteria for reporting qualitative research [43].

In this study we interviewed 17 participants, which was considered a substantial number of participants in a qualitative study. Sample size differs in qualitative studies, but it is always small [44]. However, by also including focus group interviews in the data collection we could have revealed broader and more nuanced perspectives. Another limitation is that three of the interviews were performed with spouses, which may have hindered vulnerable information to be revealed.

## Conclusion

Most of the interviewed participants expressed to suffer from the post-COVID-19 condition with various physical and cognitive symptoms, such as ‘brain fog’ approximately 6 months after hospitalisation for COVID-19. These findings were in line with larger studies.

The novel findings of this study were connected to existential topics. The patients’ expressions and reflections of guilt and gratitude, and new life perspectives were revealed. This showed their ability to engage resources to cope with stressors. The knowledge drawn from this study can be useful for health professionals to approach the older patients suffering from post-COVID-19 condition holistically and support their coping resources.

## Data Availability

The data that support the findings from this study are not publicly available due to the permission given from the Regional Committee for Ethics in Medical Research (South-East Norway).
